# The Role of Microglia in Prion Diseases: A Paradigm of Functional Diversity

**DOI:** 10.3389/fnagi.2017.00207

**Published:** 2017-06-23

**Authors:** Juliane Obst, Emilie Simon, Renzo Mancuso, Diego Gomez-Nicola

**Affiliations:** Biological Sciences, University of Southampton, Southampton General HospitalSouthampton, United Kingdom

**Keywords:** microglia, Csf1r, proliferation, neuroinflammation, neurodegnerative diseases

## Abstract

Inflammation is a major component of neurodegenerative diseases. Microglia are the innate immune cells in the central nervous system (CNS). In the healthy brain, microglia contribute to tissue homeostasis and regulation of synaptic plasticity. Under disease conditions, they play a key role in the development and maintenance of the neuroinflammatory response, by showing enhanced proliferation and activation. Prion diseases are progressive chronic neurodegenerative disorders associated with the accumulation of the scrapie prion protein PrP^Sc^, a misfolded conformer of the cellular prion protein PrP^C^. This review article provides the current knowledge on the role of microglia in the pathogenesis of prion disease. A large body of evidence shows that microglia can trigger neurotoxic pathways contributing to progressive degeneration. Yet, microglia are also crucial for controlling inflammatory, repair and regenerative processes. This dual role of microglia is regulated by multiple pathways and evidences the ability of these cells to polarize into distinct phenotypes with characteristic functions. The awareness that the neuroinflammatory response is inextricably involved in producing tissue damage as well as repair in neurodegenerative disorders, opens new perspectives for the modulation of the immune system. A better understanding of this complex process will be essential for developing effective therapies for neurodegenerative diseases, in order to improve the quality of life of patients and mitigating the personal, economic and social consequences derived from these diseases.

## Microglia in The Healthy Brain

### Origin and Turnover of Microglia

Since the initial description of microglial cells by Pio Del Rio Hortega (del Río-Hortega, 1920, 1932; del Río-Hortega and Penfield, 1927), their origin has been a source of debate. However, it has been recently established that tissue resident macrophages as microglia originate from erythroid-myeloid progenitors (EMPs) emerging from the yolk sac (YS) during primitive hemeatopoiesis at embryonic stages 7.0 (E7.0) to E9.5 (Cuadros et al., 1993; Alliot et al., 1999; Schulz et al., 2012; Gomez Perdiguero et al., 2015; Sheng et al., 2015; Wang et al., 2015). A pioneering study by Ginhoux et al. (2010) allowed finding the earliest microglial progenitors in the YS during mouse development, thanks to fate-mapping experiments that allowed tagging early YS blood-island cells and then follow the emergence of microglial cells into the central nervous system (CNS). Fate mapping of YS progenitors from E6.5 to E7.0 produced tagging of less than 4% of adult microglia, whereas mapping from E7.0 to E7.25 produced 29% microglia being labeled, allowing the definition that primitive EMPs that arise before E7.5 are the main contributors to the adult microglial population (Ginhoux et al., 2010). Then, several studies using tamoxifen-inducible Cre lines in which the Cre-ER-T2/Mer-Cre-Mer protein was expressed under the control of different genes such as Colony stimulating factor receptor 1 (*Csf1r*; Schulz et al., 2012), *C-kit* (Sheng et al., 2015), TEK Receptor Tyrosine Kinase (*Tie2*; Gomez Perdiguero et al., 2015) corroborated that microglial progenitors have a YS origin. A recent study by Mass et al. (2016) has allowed a more precise definition of the sequence of differentiation steps leading to the adult microglial population. In the YS, uncommitted EMPs (Kit^+^ CD45^lo^ Csf1r^+^ AA4.1^+^) differentiate into pre-macrophage (pMac; kit^−^ CD45^hi^ F4/80^−^) that do not yet have a microglial phenotype. From E9.5, as they initiate a core macrophage transcriptional program, those pMacs colonize the whole embryo in a C-X3-C motif chemokine receptor 1 (CX3CR1)-dependent manner. Indeed, at E9.5 and E10.5, CX3CR1-deficient embryos exhibit a delay in the colonization of progenitors and a decrease of pMacs and macrophages population in the head while they display an accumulation of pMacs in the YS and fetal liver (Mass et al., 2016). Immediately following colonization of the embryonic brain, a tissue specific transcriptional program is triggered and leads to the production of postnatal microglia, including a downregulation of T-cell immunoglobulin and mucin domain containing 4 (*Timd4*) and mannose receptor (*Cd206*) and an upregulation of Sal-like (*Sall*)*1* and *Sall3* (Lavin et al., 2014; Mass et al., 2016). As the embryo develops, microglia progenitors mature in a Interferon regulatory factor 8 (IRF8) and PU.1-dependant manner by expressing a set of different markers including CSF1R, Runt-related transcription factor (Runx1), ionized calcium-binding adaptor molecule 1 (Iba1), C-X3-C Motif Chemokine Receptor (CX3CR1), Tie2, the cluster of differentiation 45 (CD45) or C-kit (Kierdorf et al., 2013; Mass et al., 2016).

In humans, microglial cells are identified in the extracerebral mesenchyme around 4.5 gestational weeks. At 5 gestational weeks, they invade the parenchyma by entering the brain primordium via the developing meninges, ventricular zone and choroid plexus (Monier et al., 2006; Verney et al., 2010) and they only exhibit a ramified morphology around the 35th week (Hutchins et al., 1990; Esiri et al., 1991; Rezaie and Male, 1999).

**Figure 1:**
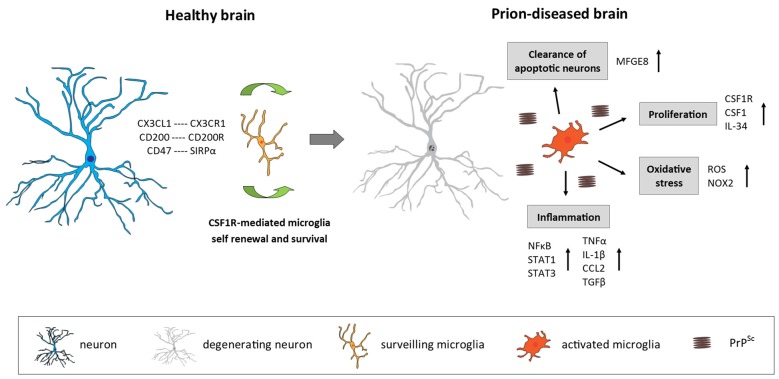
Microglia in the prion-diseased brain. In the healthy brain, microglia constantly surveil their microenvironment for any disturbance of brain homeostasis and are kept in this surveiling state by signals mainly originating from healthy neurons. The microglial population is maintained by local self-renewal mediated via CSF1R and its ligands CSF1 and IL-34. In the context of neurodegeneration and protein accumulation present in the prion-diseased brain, the microglial response is characterized by functional changes involving increased proliferation, an inflammatory activation and the removal of apoptotic neurons. CX3CL1, C-X3-C motif chemokine ligand 1; CX3CR1, C-X3-C motif chemokine receptor 1; CD200, Cluster of differentiation 200; CD47, Cluster of differentiation 47; SIRPα, Signal regulatory protein α; CSF1R, Colony stimulating factor 1 receptor; CSF1, Colony stimulating factor; IL-34, Interleukin 34; MFGE8, Milk fat globule-EGF factor 8 protein; ROS, Reactive oxygen species; NOX2, Nicotinamide adenine dinucleotide phosphate-oxidase (NADPH) oxidase 2; NFκB, Nuclear factor kappa-light-chain-enhancer of activated B cells; STAT1/3, Signal transducer and activator of transcription 1/3; TNFα, Tumor nerosis factor α; IL-1β, Interleukin 1β; CCL2, C-C motif chemokine ligand 2; TGFβ, Transforming growth factor β; PrP^Sc^ misfolded prion protein (scrapie).

In the adult, the microglial population is maintained by a self-renewal process (Lawson et al., 1992; Askew et al., 2017; Figure 1). A foundational study by Lawson et al. (1992) defined a remarkably slow turnover rate of adult murine microglia, by means of analyzing short-term ^3^H tymidine incorporation. However, more recent insight arising from repopulation paradigms suggested that microglia could have a higher turnover capacity in the steady state. The pharmacological depletion of microglia, by using a potent CSF1R inhibitor, is followed by the rapid reconstitution of the microglia population by proliferation of resident microglia, without the contribution of circulating monocytes (Elmore et al., 2014). A transgenic paradigm, allowing the depletion of microglia by diphtheria toxin injection, validated this repopulating capacity, independent of circulating monocytes (Bruttger et al., 2015). Although these repopulation paradigms are distal from modeling a homeostatic system, they suggested a latent potential for microglial cells to proliferate more rapidly than thought before, allowing the colonization of an empty niche. In this line, a recent study from our group showed that in mice and humans the turnover of microglia in the steady state is remarkably fast, allowing the whole population to be renewed several times during a lifetime (Askew et al., 2017). Indeed, whereas the previous study in the healthy brain, using ^3^H thymidine and immunohistochemistry for F4/80, had shown that, at a given time, only 0.05% of the microglia were proliferating (Lawson et al., 1992), Askew et al. (2017) recently demonstrated that 0.69% of the microglial population is proliferating by using more sensitive techniques (BrdU incorporation detected in Iba1^+^ cells and 2-photon live imaging). Microglial proliferation is balanced by microglial apoptosis, with these two mechanisms being synchronized in time and space, allowing for a rapid remodeling of the microglial landscape during a lifetime, without the contribution of circulating monocytes (Askew et al., 2017).

### Distribution and Function of Microglia in the Adult Brain

The density and morphology of microglia varies considerably across the healthy adult brain. In the mouse brain, there are an estimated total number of 3.5 × 10^6^ microglial cells, however their distribution varies from 5% in the cortex and corpus callosum, to 12% in the substantia nigra (Lawson et al., 1990). In mice, microglia are more numerous in gray matter than white matter and areas as the hippocampus, basal ganglia and substantia nigra are particularly densely populated in microglia (Lawson et al., 1990). In comparison, the less densely populated areas include fiber tracts, cerebellum and much of the brainstem whereas the cerebral cortex, thalamus and hypothalamus have average cell densities (Lawson et al., 1990). This cell density remains constant from early postnatal development to aging, thanks to the constant turnover of the population (Askew et al., 2017). In the human brain, microglia has been estimated to make up 6%–18% of neocortical cells (Mittelbronn et al., 2001; Pelvig et al., 2008; Lyck et al., 2009). Similarly, microglial morphology varies considerably depending on specific regional properties. Whereas they are usually more ramified in gray matter, in white matter they display elongated somata and less ramified processes preferentially oriented along fiber tracts (Lawson et al., 1990; Mittelbronn et al., 2001; Torres-Platas et al., 2014). The molecular determinants of those anatomical differences in diversity and morphology are not clearly defined, however recent studies showed that microglia have a distinct region-dependent and age-dependent transcriptomic signature corroborating the existence of the regional heterogeneity of microglial phenotypes (Hickman et al., 2013; Grabert et al., 2016; Soreq et al., 2017).

In the healthy brain, microglia display a “surveilling” phenotype characterized by a small cell body and long branching processes serving to continuously sense the microenvironment to detect any alteration of CNS homeostasis (Kettenmann et al., 2013; Figure 1). Microglia show invariant soma positions but continually and rapidly moving processes (average velocity around 2.5 μm/min; Davalos et al., 2005; Nimmerjahn et al., 2005; Wake et al., 2009). Microglias rapidly change their surveilling phenotype into a diversity of “activated” phenotypes after the alteration of the CNS homeostasis or presence of a threat to neuronal integrity. They adopt a more amoeboid and less ramified phenotype with a large soma and rapidly trigger appropriate responses, which could range from upregulation or *de novo* synthesis of cell-surface molecules such as CD68 and major histocompatibility complex (MHC) class II to phagocytosis or the release of molecular mediators, including immune and non-immune factors (Kettenmann et al., 2011; Figure 1). Consequently, this activated phenotype is a generalized term that fails to take into account that microglia can adopt numerous functionally different phenotypes, depending on the exact nature of the stimulus (Perry et al., 2010).

In the healthy brain, microglia is involved in several mechanisms regulating CNS physiology. Microglia are in direct contact with dendritic spines, axons and synapses, suggesting that they participate in the regulation of synaptic structure and function (Wake et al., 2009; Paolicelli et al., 2011; Squarzoni et al., 2014). One of these mechanisms is synaptic pruning, a process by which excess synapses formed in the developing brain are eliminated to thereby increase the efficiency of the neural network. In this process, microglia has been shown in direct contact with the synapses and removing unwanted elements by phagocytosis (Paolicelli et al., 2011). Electron microscopy and high resolution *in vivo* engulfment assays have showed also the presence of presynaptic and postsynaptic elements inside microglial lysosomes (Berbel and Innocenti, 1988; Tremblay et al., 2010; Schafer et al., 2012). Some proteins as complement receptor 3 (CR3/CD11b), CX3CR1 or the adaptor protein DNAX-activating protein of 12-kDa (DAP12), highly expressed by microglia, are involved in the process of synaptic pruning. Indeed it has been shown that disruption of one of those proteins resulted in synaptic abnormalities in both prenatal and postnatal brain development (Stevens et al., 2007; Paolicelli et al., 2011; Squarzoni et al., 2014).

As mentioned above, the interactions between microglia and their surrounding cells have a major role in the determination of the microglial phenotype. Healthy neurons maintain microglia in their surveilling state via secreted and membrane bound signals. The interaction of neuronal CD200 with microglial CD200R leads to inactivation of microglia and plays a critical role in neuroprotection (Lyons et al., 2007). In CD200-deficient mice, microglia are more numerous, form more aggregate-like structures, display less ramifications with shorter processes and show an upregulation of CD45 and CD11b, which are markers of activation (Hoek et al., 2000). The bidirectional signaling between SIRPα and CD47 that can be co-expressed by both neurons and microglia maintains microglia in their surveilling state by inhibiting phagocytosis and inducing the synthesis of the anti-inflammatory cytokines (Zhang et al., 2015). The interaction of neuronal CX3CL1 with microglial CX3CR1 constrains microglial activation (Lyons et al., 2009). In models for Parkinson’s disease (PD) under a CX3CR1-deficient background, microglia exhibit an over-activated phenotype and neuronal cell death is enhanced (Bhaskar et al., 2010; Cho et al., 2011). The neurotoxicity induced by activated microglia in neurodegenerative diseases seems to be worsened in CX3CR1-deficient mice, suggesting that the signaling through CX3CL1/CX3CR1 regulates the phenotype of microglia (Cardona et al., 2006). Another receptor involved in the maintenance of the microglial surveilling state is the triggering receptor expressed on myeloid cells 2 (TREM2) associated with the adaptor protein DAP12. TREM2 is essential for phagocytosis process by microglia (Takahashi et al., 2007) and has recently been shown to interact with specific lipids to promote microglial survival (Wang et al., 2015). Mutations leading to a loss of function in TREM2 or DAP12 underlie the Nasu–Hakola disease, in which patients display progressive presenile dementia (Paloneva et al., 2000, 2002).

Microglia also express receptors that trigger essential cellular survival and developmental signals. CSF1R plays a major role in microglial development and survival. Expressed by microglia (Akiyama et al., 1994; Raivich et al., 1998), CSF1R is activated by two homodimeric glycoprotein ligands, CSF1 (Stanley and Heard, 1977) and Interleukin 34 (IL-34; Lin et al., 2008). In the brain, IL-34 is primarily expressed by neurons (Mizuno et al., 2011; Wang et al., 2012) whereas CSF1 is mainly expressed by microglia (Chitu et al., 2016). These two ligands present different patterns of regional expression in the prenatal and postnatal brain. CSF1 is highly expressed in the neocortex, corpus callosum, cerebellum and spinal cord, whereas IL-34 is highly expressed in the forebrain (neocortex, olfactory bulb and striatum; Wei et al., 2010; Nandi et al., 2012). The binding of CSF1 or IL-34 to CSF1R leads to the oligomerization and transphosphorylation of CSF1R followed by the phosphorylation and activation of downstream cytoplasmic mediators that promote microglia development, survival and proliferation (Ségaliny et al., 2015). As described previously, the development of tissue-resident macrophages including microglia is dependent on *Csf1r* expression from the first stages of development (Mass et al., 2016). Moreover, IL-34-deficient and CSF1-deficient mice display fewer microglia in various regions of the brain while Csf1r-deficient mice are completely devoid of them (Dai et al., 2002; Ginhoux et al., 2010).

In addition to their manifold functions in maintaining homeostasis in the healthy brain, microglia have been shown to play a major role in driving innate inflammatory responses in many neurodegenerative diseases. Prion disease is characterized by progressive neurodegeneration, which is accompanied by a pronounced microglia-mediated immune response, therefore being an extraordinary model to study the role of microglia in chronic neurodegenerative diseases. In this review, we will explore the molecular determinants of the contribution of microglia to the pathogenic cascade in prion disease, aiming to address some of the most relevant remaining unknowns of the role of microglia in prion disease: is microglial activation merely a bystander effect of prion pathology, or what aspects of microglia-mediated immune response are contributing to disease outcome in a beneficial or detrimental manner? Can the microglia-derived inflammatory response directly harm neurons and lead to neuronal degeneration, or is neuronal loss a consequence of misfolded prion protein (scrapie) (PrP^sc^) accumulation, or a combination of both? In the following sections, we will provide a comprehensive picture defining many aspects of microglial biology in prion disease.

## Prion Pathology

Transmissible spongiform encephalopathies or prion diseases, such as Creutzfeldt-Jakob disease (CJD), are fatal neurodegenerative disorders that affect humans and many other mammals (Aguzzi and Calella, 2009). The infectious agent consists of PrP^Sc^. PrP^Sc^ can aggregate, recruit and convert benign cellular prion protein (PrP^C^) into abnormal pathological isoforms (Aguzzi and Calella, 2009). Thereby, prions act as “seeds” that trigger a chain reaction of PrP misfolding and aggregation (Jarrett and Lansbury, 1993). Prion diseases have a heterogeneous etiology, as they can be genetic, infectious or sporadic. Infectivity requires the transfer of prion seeds from affected individuals into healthy hosts, whereas in genetic and idiopathic cases prion protein undergoes a spontaneous misfolding of PrP molecules into self-propagating seeds. In humans, sporadic CJD (sCJD) is the most common prion disease, followed by genetic CJD (gCJD) and transmitted CJD (iatrogenic CJD and variant CJD; Aguzzi and Calella, 2009). CJD has been also shown to be transmitted through blood or blood derivatives (Llewelyn et al., 2004; Bishop et al., 2013).

The prion neuropathology is characterized by spongiform degeneration, synaptic and neuronal loss, gliosis and the accumulation of aggregated PrP^SC^ (DeArmond and Prusiner, 1995; Cunningham et al., 2003; Wadsworth and Collinge, 2011; Hilton et al., 2013). Prion disease typically has long incubation times and a rapid disease progression, and can be manifested in different ways, with behavioral and pathological differences within and across species (Prusiner, 1998; Tanaka et al., 2006; Collinge and Clarke, 2007; Colby and Prusiner, 2011). Interestingly, different strains of prion (e.g., ME7, 79A, 22L, 22A) preferentially affect specific regions of the brain in mice (Cunningham et al., 2005a). The simplest explanation for such regional selectivity would be a differential tropism of prion strains and thereby a regional aggregation and toxicity. However, recent experiments assessing prion misfolding using highly sensitive techniques showed that prion protein seeds accumulate in all brain regions irrespective of neurodegeneration (Alibhai et al., 2016).

Prions disease also provides an interesting experimental approach to model many aspects of neurodegenerative diseases associated with protein misfolding. In mouse models of prion disease, microglia become activated early in the disease process thereby representing a valuable tool for elucidating the impact of neuroinflammation in chronic neurodegenerative disorders.

## Microglial Proliferation and Activation in Prion Disease

Prion disease is characterized by an increase in the number of microglia, associated with an activated and phagocytic phenotype (Perry et al., 2002; Perry and O’Connor, 2010; Figure 1). The relative contribution of local proliferation of microglia vs. the infiltration of bone-marrow derived progenitors to this increase has been a source of debate during recent years (Gomez-Nicola and Perry, 2015). However, a recent study demonstrated that, in a murine model of prion disease, local proliferation of resident microglial cells is a major component in the evolution of chronic neurodegeneration (Gómez-Nicola et al., 2013). The increase in microglial density and proliferative activity varies across different regions such as the hippocampus (CA1) and the thalamus, the later showing the biggest increase in cell numbers (Gómez-Nicola et al., 2013). This increase in microglial numbers is independent of the recruitment of circulating monocytes, evidenced by comparing microglial density in prion diseased mice with a C-C chemokine receptor type 2 (CCR2)^−/−^ background with WT mice (Gómez-Nicola et al., 2014).

The proliferation of microglia in prion disease is regulated by the activation of CSF1R and the transcription factors PU.1 and CCAAT/enhancer-binding protein alpha (C/EBPα, being this system also active in human variant CJD and Alzheimer’s disease (AD; Gómez-Nicola et al., 2013; Olmos-Alonso et al., 2016). The inhibition of CSF1R blocks the proliferation of microglia, leading to a decrease in neuronal death in the hippocampus (Gómez-Nicola et al., 2013). A recent study showed that prolonged inhibition of CSF1R in APPswe/PSEN1dE9 (APP/PS1) mice, a model of AD-like pathology, blocks microglial proliferation and leads to the prevention of synaptic degeneration and to an improvement of performance in memory and exploratory tasks (Olmos-Alonso et al., 2016). CSF1R blockade also showed positive effect in mutant superoxide dismutase 1 (SOD1) models of Amyotrophic Lateral Sclerosis (ALS) by reducing microglial proliferation in the spinal cord and macrophage infiltration into peripheral nerves (Martínez-Muriana et al., 2016). The studies focused to targeting CSF1R suggest that microglial proliferation in prion disease, AD and ALS has a net detrimental contribution to the disease progression.

Targeting microglial proliferation by the specific inhibition of CSF1R renders a different experimental outcome than the unspecific removal of microglia. Some studies have aimed at eliminating microglial cells in prion disease, either by the transgenic expression of thymidine kinase (TK) and “suicide” of proliferating CD11b+ cells (Zhu et al., 2016), or by the non-specific blocker of mitosis cytosine arabinoside (Ara-C; Gómez-Nicola et al., 2013). These approaches indicated a neutral or beneficial role of microglia, as their elimination did not change the trajectory of the disease. However, the technical limitations of these targeting approaches difficult the interpretation. For example, the use of CD11b-TK mice leads to a prominent and uncontrolled death of microglia in the context of on-going neurodegeneration, not providing a physiologically silent way to address the contribution of the cells. Also, the TK transgene in CD11b-TK mice is activated by the administration of ganciclovir, an agent recently identified to have a potent anti-proliferative impact on microglia during brain pathology (Ding et al., 2014). Similarly, the use of Ara-C causes a shift in the microglial phenotype towards a detrimental pro-inflammatory profile, independent from its effects on cell proliferation, accelerating neuronal death (Gómez-Nicola et al., 2013). Together, these findings suggest that the specific and selective targeting of microglial proliferation, instead to their elimination, is an optimal approach to understand the contribution of these cells to the pathology.

Activation of microglia is detectable from early stages of prion disease pathogenesis (Betmouni et al., 1996) and becomes more widespread as the disease progresses, closely associated with the spread of neurodegeneration (Perry, 2016). Microglial activation appears simultaneously with first behavioral deficits (Guenther et al., 2001), at a time point when synapses start to degenerate in the stratum radiatum of the hippocampus, but no neuronal loss occurs yet (Cunningham et al., 2003; Gray et al., 2009). Whether microglia activation is directly caused by accumulating misfolded PrP^sc^ or as a response to synaptic damage cannot be reliably concluded. While studies *in vitro* have demonstrated that microglia can be activated directly by PrP and subsequently damage neurons (Brown et al., 1996; Giese et al., 1998), there is limited evidence of a direct response to PrP^Sc^ aggregates *in vivo*. In contrast, the mere presence of misfolded prion protein, as detected in various brain regions using high sensitivity techniques, might not be sufficient to induce a microglia-mediated immune response in all brain regions (Alibhai et al., 2016). However, it is widely accepted that microglia activation precedes neuronal degeneration and the onset of clinical disease (Williams et al., 1997; Giese et al., 1998).

The cytokine profile in the prion-diseased brain is associated with the expression of both pro- and anti-inflammatory molecules (Perry, 2016; Figure 1). While a number of studies demonstrated a profile shifted to the anti-inflammatory spectrum, dominated by the expression of transforming growth factor β (TGFβ, C-C Motif Chemokine Ligand 2 (CCL2) and prostaglandin E2 (PGE2) with a limited pro-inflammatory response characterized by IL-1β and tumor necrosis factor α (TNFa; Minghetti et al., 2000; Walsh et al., 2001; Cunningham et al., 2002, 2005b; Perry et al., 2002), other studies have reported that also pro-inflammatory factors are up-regulated in the prion brain (Campbell et al., 1994; Williams et al., 1994; Kordek et al., 1996), suggestive of a mixed inflammatory profile. The lack of consensus regarding the inflammatory profile in the prion brain may arise from the fact that different prion strains, stages of disease and techniques of detection were used. Recent studies using a broader panel of markers support the hypothesis that both pro-and anti-inflammatory factors contribute to the immune response in prion disease. Vincenti et al. (2015) re-analyzed a large transcriptomic database of brains from multiple mouse strains exposed to various prion strains and collected at different stages of disease progression (Hwang et al., 2009) and proposed that most of the differentially expressed genes in the prion brain were of microglial origin and associated with the inflammatory response. Microglia isolated from 79A-infected mice showed increased expression of IL-1β, TNFα and CSF1, but not IL-6, IL-10 or TGFβ, which correlates with disease progression and indicates a classical activation phenotype of microglia in this prion model (Vincenti et al., 2015). A recent longitudinal study reported new inflammatory genes upregulated early in the prion brain, including genes involved in inflammation, monocyte recruitment and growth regulation (Carroll et al., 2015). Concerning signal transduction pathways, an early activation predominantly of the Signal transducer and activator of transcription (STAT)- and Nuclear factor kappa-light-chain-enhancer of activated B cells (NFkB) pathways has been observed in prion disease models, determined by the up-regulation of STAT- and NFkB-responsive genes, including many cytokines and chemokines, as well as by the detection of increased phosphorylation of STAT1 and STAT3 specifically (Llorens et al., 2014; Carroll et al., 2015). The inflammatory response seems to be quite consistent between different murine prion strains. Although prion strains 22L, RML and ME7 revealed cellular and regional differences in PrP^Sc^ accumulation, they showed a similar up-regulation mainly of pro-inflammatory genes and chemokines which correlated with the deposition of PrP^Sc^ and the onset of glial activation (Carroll et al., 2016).

Targeting individual immune pathways which are dysregulated in prion disease have shown differential effects in modifying pathology. The absence of CCL2 does not drastically affect the disease course (Felton et al., 2005; O’Shea et al., 2008) and similarly, reducing PGE2 levels using dapsone or Nonsteroidal anti-inflammatory drugs (NSAIDs) did not impact disease progression (Guenther et al., 2001; Perry, 2010). On the contrary, inhibition of TGFβ enhanced neurodegeneration, indicating that this immune mediator is critically involved in regulation of the innate immune response in prion disease (Boche et al., 2006). TNFα as well as TNF receptor 1 knockout mice show normal prion disease progression after intracerebral injection of the prion protein (Klein et al., 1997; Mabbott et al., 2000), whereas deficiency of IL-1 receptor type 1 prolongs prion incubation time (Tamgüney et al., 2008) and delays disease onset and protein aggregation and increases survival of diseased mice (Schultz et al., 2004). PrP fibrils have been found to induce IL-1β secretion by microglia *in vitro*, dependent on components of the Nod-like receptor family pyrin domain containing 3 (NLRP3) inflammasome, is sufficient to induce neuronal toxicity (Hafner-Bratkovič et al., 2012; Shi et al., 2012). However, an *in vivo* study using mice deficient in NLRP3 or the adaptor protein apoptosis-associated speck-like protein containing a caspase recruitment domain (ASC) and thereby lacking functional NLRP3 inflammasome, failed to demonstrate a significant impact on prion pathogenesis, indicating that inflammasomes do not contribute to prion progression (Nuvolone et al., 2015).

Numerous studies have reported signs of oxidative stress in brains of CJD patients as well as in murine models of prion disease (Guentchev et al., 2002; Van Everbroeck et al., 2004; Yun et al., 2006), which might play a role in disease progression by contributing to neurotoxicity and neurodegeneration. Stimulation of microglia with PrP fragments *in vitro* has been shown to induce growth arrest and the release of nitric oxide (NO) and PGE2 (Villa et al., 2016). Furthermore, in patients with CJD as well as in a mouse model of prion, production of NADPH oxidase 2 (NOX2) was up-regulated specifically by microglia in the affected brain regions (Sorce et al., 2014). Prion-induced mice deficient in NOX2 demonstrated decreased production of reactive oxygen species (ROS), a delayed onset of motor deficits and an increased survival time, indicating that microglia-specific NOX2 production leads to the release of ROS and affects prion pathology (Sorce et al., 2014).

It has been shown that microglia are able to engulf and clear apoptotic neurons (Hughes et al., 2010; Kranich et al., 2010). The phagocytic function of microglia is increased in the prion-diseased brain and associated with enhanced expression of scavenger receptors, cathepsins, and proteins of the respiratory burst, while phagocytic microglia were characterized by a lack of IL-1β expression (Hughes et al., 2010). While microglia were efficient in the uptake of injected latex beads and apoptotic cells, they were unable to remove prion protein aggregates, even upon additional stimulation with lipopolysaccharide (LPS; Hughes et al., 2010). Phagocytosis of apoptotic neurons by microglia has been shown to be dependent on milk fat globule epidermal growth factor 8 (MFGE8). Ablation of mfge8 resulted in accelerated prion pathology, with reduced clearance of apoptotic bodies and increased prion protein accumulation, indicating that microglia phagocytosis via MFGE8 is a protective mechanism in prion disease (Kranich et al., 2010). While *in vitro* studies using microglia cells or organotypic brain slices proposed that microglia can clear PrP and thereby decrease prion titers (McHattie et al., 1999; Falsig et al., 2009; Kranich et al., 2010; Zhu et al., 2016), evidence that they do so *in vivo* is still missing. It is possible that the removal of misfolded prion protein is simply inefficient or that PrP^sc^ is not a sufficient trigger to induce phagocytosis, a similar concept to that proposed for phagocytosis of amyloid β (Aβ in AD (Guillot-Sestier and Town, 2013; Prokop et al., 2013). Furthermore, while the phagocytic function of microglia in prion disease is mostly considered to be beneficial and protective, it is also conceivable that microglia, by taking up cell debris from prion infected cells or possibly PrP aggregates, might even contribute to the spreading of the pathogenic protein (Baker et al., 2002).

TREM2 has been implicated in several neurodegenerative diseases such as AD (Jonsson et al., 2012; Guerreiro R. J. et al., 2013), frontotemporal dementia (Guerreiro R. et al., 2013) and ALS (Cady et al., 2014). TREM2 is an innate immune cell receptor expressed on microglia and other myeloid cells is thought to be involved in phagocytosis of apoptotic neurons and promoting an anti-inflammatory phenotype (Takahashi et al., 2005, 2007; Hsieh et al., 2009). In the context of prion disease, TREM2 has been demonstrated to be up-regulated after prion infection, but the depletion of TREM2 did neither change incubation time and survival, nor microglia immune phenotype during prion disease (Zhu et al., 2015).

Recently, there has been evidence of the involvement of non-coding microRNAs (miR) in regulating the microglia inflammatory response in prion disease. A number of miRs implicated in the regulation of gliosis, glial cell proliferation, the innate-immune response, inflammatory signaling, deficits in neurotrophic signaling and synaptogenesis have been found to be upregulated in human prion disease cases (Zhao et al., 2016). Among them, miR-146a was observed to influence immune response and activation state of microglia (Saba et al., 2012).

While increasing evidence indicates that microglia activation seems to contribute to prion pathogenesis, not much is known about the mechanistic underpinnings. We have shown recently that microglial expansion negatively affects prion disease pathology (Gómez-Nicola et al., 2013), pointing towards a net detrimental role of microglia during chronic neurodegeneration. Another recent study emphasized a protective role of microglia in prion disease, demonstrating accelerated disease pathology upon microglia ablation in the brain (Zhu et al., 2016). This dual role of microglia during prion disease provides further proof that microglia function is highly dynamic and versatile, by promoting neurotoxic effects through facilitating a potentially aberrant and harmful inflammation in prion disease, but also by provoking a protective response through tissue maintenance and repair.

## Systemic Inflammation and Microglial Priming

The study of immune to brain communication is gaining interest, with the immune system as a transducer of both endogenous and exogenous challenges to the host. It is known that systemic infection and inflammation are able to produce behavioral changes, known as “sickness behaviors”, that include fever, malaise, anorexia, lethargy and depression (Dantzer et al., 1998), demonstrating an important influence of the immune system on CNS processes. LPS challenge in healthy humans has been shown to produce sickness behavior with fever and neuropsychological symptoms, including reduced declarative memory performance (Krabbe et al., 2005) and increased symptoms of depression (DellaGioia et al., 2012). Systemic inflammation communicates with the brain by several neural and humoral pathways. Receptors for inflammatory mediators present on vagus nerve fibers can respond to inflammatory signals and inform the nucleus of the solitary tract and other regions of the brain (Wang et al., 2003). Macrophages in the circumventricular organs (CVOs), which are regions of the CNS without a tight blood-brain barrier (BBB), are also able to communicate with systemic inflammatory mediators. Signals generated in the CVOs subsequently travel to other regions of the brain (Lacroix et al., 1998). A third route of communication involves cerebral endothelial cells of the BBB, which can transduce signals from blood to the CNS (Laflamme and Rivest, 1999). The molecular pathways by which systemic inflammation alters brain function leading to sickness behavior are not completely elucidated. It has been suggested that the link between the systemic immune system and the CNS is located at the level of the hypothalamus, the main brain region controlling the neuroendocrine system. Systemic inflammation or ageing can modify the hypothalamic function though NFkB activation and microglial-neuronal crosstalk (Li et al., 2012; Zhang et al., 2013). Further studies are needed to elucidate the exact mechanism underpinning the alteration of neuronal function by systemic inflammation.

It is known that microglia play an important role in immune-to-brain communication (Perry and Teeling, 2013). Within this context, the concept of microglial priming has been proposed. Microglial priming occurs as a result of damage associated with chronic neurodegenerative conditions (e.g., misfolded protein, neuronal debris or vascular changes), or several other stressors affecting the nervous system, such as maternal separation, acute injury or aging. It consists of an exaggerated or heightened microglial response—much stronger than that observed in stimulus-naïve microglia—to a second inflammatory stimulus. Most of the evidence supporting the hypothesis of microglial priming in chronic neurodegenerative conditions arises from the study of prion models. In fact, microglial priming was first described in prion brains subjected to a systemic challenge mimicking systemic infection (Combrinck et al., 2002). Cunningham et al. (2005b) showed how intracerebral LPS injection in prion mice results in exacerbated IL-1β expression, neutrophil infiltration and NO expression, accompanied with increased cell death. Systemic injection of LPS (Cunningham et al., 2009), Polyinosinic-polycytidylic acid (poly I:C; Cunningham et al., 2002, 2005b, 2007; Field et al., 2010) or TNFα (Hennessy et al., 2017) in prion mice also leads to exacerbated in the brain, with increased expression of pro-inflammatory cytokines and chemokines including IL-1β TNFα and CCL2. Interestingly, the increased inflammation triggered by LPS in ME7 prion brain is independent on circulating IL-1β and IL-6 (Murray et al., 2011; Hennessy et al., 2017). Although the exact mechanism by which microglia is primed is still unknown, it has been suggested that cyclooxygenase 1 (COX-1) expression in microglia mediates the systemic effects of LPS in the prion brain (Griffin et al., 2013). More recently, Hennessy et al. (2017) reported that astrocytes could be also primed in the prion brain, and generate exaggerated levels of cytokines and chemokines after systemic LPS challenge. Although these findings suggest that microglia in the prion diseased brain are primed by the ongoing pathology and that a secondary stimulus switches these cells to an aggressive phenotype, there is still no defining criteria to classify this state, and it remains unclear whether different patterns of priming result from different forms of neurodegeneration or different systemic inflammatory stimuli.

Interestingly, priming observations and the impact of the immune system on the CNS are not restricted to prion disease (Perry, 2010; Cunningham, 2013). Systemic LPS injection can induce degeneration of cells in the substantia nigra (Gao et al., 2002; Qin et al., 2007) and systemic challenge with IL-1β in an animal model of PD leads to enhanced degeneration of substantia nigra neurons and pro-inflammatory cytokine production (Pott-Godoy et al., 2008). Systemic challenge with LPS or other bacterial toxins in an experimental model of multiple sclerosis, Experimental autoimmune encephalomyelitis (EAE), can also exacerbate neurological symptoms (Schiffenbauer et al., 1993). Systemic LPS challenge caused increased synthesis of pro-inflammatory cytokines in the CNS of transgenic mouse models of AD-like pathology (Sly et al., 2001), and tau phosphorylation (Roe et al., 2011). Chronic systemic inflammation in the form of osteoarthritis results in accelerated neuroinflammation and Aβ pathology in APP/PS1/Col1-IL1βXAT mice (Kyrkanides et al., 2011). There is also evidence about the impact of the immune system upon human neurodegenerative diseases. Systemic infection and increased systemic inflammation have been associated to an enhanced cognitive decline in AD patients (Holmes et al., 2003, 2009, 2011), and systemic infections are associated with relapses in multiple sclerosis in patients (Buljevac et al., 2002).

Overall, evidence suggests that systemic inflammation contributes to the pathology during chronic neurodegenerative conditions, with microglia acting as a hub of communication between the systemic and CNS compartments. According to this view, it is likely that a correct treatment and management of systemic inflammation could potentially delay the progression of neurodegenerative disorders.

## Role of Microglia in Human Prion Diseases

Evidence from the literature supports a role of microglia-mediated inflammation in human prion diseases. Activated microglia are found in the brains of CJD patients (Sasaki et al., 1993; Szpak et al., 2006) where they appear to be closely associated with PrP^Sc^ deposits (Miyazono et al., 1991; Guiroy et al., 1994; Muhleisen et al., 1995). However the degree of microglial reactivity seems to depend on the subtype of prion disease and the type of biochemical PrP^Sc^ (Puoti et al., 2005; Shi et al., 2013). Increased levels of inflammatory cytokines such as IL-8, CCL2, TGFβ, TNFα and IL-1β have been found in the cerebrospinal fluid (CSF) of sporadic CJD cases (Sharief et al., 1999; Stoeck et al., 2006, 2014) and the inflammatory response seems to correlate with the severity of lesions (Van Everbroeck et al., 2002). A recent study demonstrated subtype-specific and region-specific changes in glia activation and the expression of inflammatory mediators in CJD, with inflammation being pre-dominant in the cerebral cortex in the CJD subtype *PRNP* codon 129 Met/Met type 1 (MM1) and in the cerebellum in *PRNP* codon 129 Val/Val type 2 (VV2) cases (Llorens et al., 2014). Microglia markers such as CD11b, Iba-1 and CD68 were found to be up-regulated in a region- and subtype-specific manner which correlated with the up-regulation of pro- and anti-inflammatory cytokines, members of the complement system, the integrin family and C-type lectin/C-type lectin-like domain (CTL/CTLD) superfamily, toll-like receptors, colony-stimulating factors and cathepsins (Llorens et al., 2014). Additionally, regulatory proteins IL-34, PU.1 and C/EBPα involved in microglial proliferation are increased in variant CJD (Gómez-Nicola et al., 2013). The increased inflammatory response observed in the two subtypes of CJD was further associated with an activation of NFkB and STAT1, 3 signaling pathways (Llorens et al., 2014). The finding that key regulators of inflammationCOX-1/2 and PGE2, are elevated in brains of CJD (Minghetti et al., 2000; Deininger et al., 2003; Llorens et al., 2014) further speaks for a crucial role of inflammatory processes in human prion disease, however the exact role of microglia-mediated inflammation during the course of the fatal disease remains to be elucidated.

## Conclusion

Increasing evidence highlights the major contribution of the expansion and activation of microglia to the pathogenesis of prion disease. Activated microglia adopts a variety of functionally diverse phenotypes depending on the disease stage and systemic influences. While their response is manifold during prion disease, targeting the microglia-mediated immune response appears to be a useful approach to modify the disease course. Determining the exact mechanistic underpinnings of the neuroinflammatory processes in prion disease is an informative step in order to develop novel treatment strategies targeting neurodegenerative disease.

## Author Contributions

JO, ES and RM jointly wrote the manuscript and contributed to the drafting. DG-N designed the contents of the revision and drafted the manuscript.

## Conflict of Interest Statement

The authors declare that the research was conducted in the absence of any commercial or financial relationships that could be construed as a potential conflict of interest.

## Abbreviations

Aβ: amyloid-β
AD: Alzheimer’s disease
ALS: amyotrophic lateral sclerosis
APP/PS1: APPswe/PSEN1dE9
Ara-C: cytosine arabinoside
ASC: apoptosis-associated speck-like protein containing a caspase recruitment domain
BBB: blood-brain barrier
CCL2: C-C motif chemokine ligand 2
CCR2: C-C chemokine receptor type 2
CD: Cluster of differentiation
C/EBPα: CCAAT/enhancer-binding protein alpha
COX: cyclooxygenase
CVO: circumventricular organs
CJD: Creutzfeldt-Jakob disease
CNS: Central nervous system
CR3: complement receptor 3
CSF: cerebrospinal fluid
CSF1: Colony stimulating factor
CSF1R: Colony stimulating factor 1 receptor
CTL/CTLD: C-type lectin/C-type lectin-like domain
CX3CL1: C-X3-C motif chemokine ligand 1
CX3CR1: C-X3-C motif chemokine receptor 1
DAP12: DNAX-activating protein of 12-kDa
EAE: Experimental autoimmune encephalomyelitis
EMP: Erythroid-myeloid progenitors
Iba1: Ionized calcium-binding adaptor molecule 1
IL-1β: Interleukin 1β
IL-34: Interleukin 34
IRF8: Interferon regulatory factor 8
LPS: lipopolysaccharide
MFGE8: Milk fβt globule-EGF factor 8 protein
MHC: Major histocompatibility complex
miR: microRNA
NFκB: Nuclear factor kappa-light-chain-enhancer of activated B cells
NLRP3: Nod-like receptor family pyrin domain containing 3
NSAID: Nonsteroidal anti-inflammatory drug
NO: nitric oxide
NOX2: Nicotinamide adenine dinucleotide phosphate-oxidase (NADPH) oxidase 2
PD: Parkinson’s disease
PGE2: prostaglandin E2
pMac: pre-macrophage
poly I:C: Polyinosinic-polycytidylic acid
MM1: *PRNP* codon 129 Met/Met type 1
PrP^C^: cellular prion protein
PrP^Sc^: misfolded prion protein (scrapie)
ROS: Reactive oxygen species
Runt1: Runt-related transcription factor 1
Sall1/3: Sal-like 1/3
SIRPα: Signal regulatory protein α
SOD1: superoxide dismutase 1
STAT1/3: Signal transducer and activator of transcription 1/3
Tie2: TEK receptor tyrosine kinase
TGFβ: Transforming growth factor β
TK: thymidine kinase
TIMD4: T-cell immunoglobulin and mucin domain containing 4
TNFα: Tumor nerosis factor α
TREM2: triggering receptor expressed on myeloid cells 2
VV2: *PRNP* codon 129 Val/Val type 2
YS: yolk sac.
